# Enhanced ECG signal based on heart rate variability: validation of the direct TR correction sequence in diabetes

**DOI:** 10.3389/fcvm.2026.1899142

**Published:** 2026-08-14

**Authors:** Shanglin Yang, Hongbin Zhou, Xuwei Liao, Yuyang Lin, Juin J. Liou

**Affiliations:** 1 School of Electrical and Information Engineering, North Minzu University, Yinchuan, Ningxia, China; 2 Microelectronics and Solid-State Electronics Device Research Center, North Minzu University, Yinchuan, Ningxia, China

**Keywords:** autonomic dysfunction, DTRC sequence, health monitoring, HRV, type 2 diabetes mellitus

## Abstract

**Background:**

Heart rate variability (HRV) is a critical biomarker for assessing autonomic dysfunction, particularly in elderly and type 2 diabetes mellitus (T2DM) populations. However, traditional HRV indices, derived from R-R intervals (RRIs), often fail to detect subtle autonomic dysfunction in complex clinical scenarios. This study proposes a novel Direct TRI Correction (DTRC) sequence to optimize HRV assessment by directly extracting TR intervals (DTRI) and applying a single-step correction. The aim is to enhance accuracy in evaluating HRV, particularly in elderly and T2DM cohorts, where early detection is critical for preventing cardiovascular complications.

**Methods:**

The study enrolled 124 participants (60 T2DM patients and 64 healthy controls) stratified into three groups based on glycemic control. HRV indices were derived from traditional RRI sequences, two-step corrected TRC sequences, and the novel single-step corrected DTRC sequences, respectively. Statistical analyses were performed on the HRV indices of three sequences to evaluate the comparative performance of DTRC sequences.

**Results:**

DTRC-based HRV indices demonstrated superior sensitivity and discriminative capability compared to both RRI-based and TRC-based HRV indices. Significant improvements were observed in RMSSD (*p* = 0.007), pNN50 (*p* = 0.043), SDSD (*p* = 0.007), LHR (*p* = 0.016), SSR (*p* = 0.010), BEI (*p* = 0.002), and MSE_LS_ (*p* = 0.009). Visualization analysis and ROC curves confirmed clearer inter-group separation, particularly for T2DM patients with poor glycemic control.

**Conclusions:**

The DTRC sequence significantly enhances HRV assessment accuracy, offering a reliable tool for early detection of autonomic dysfunction in high-risk populations. Its simplified correction process and improved sensitivity hold promise for clinical diagnostics and wearable health monitoring applications.

## 1 Introduction

Heart rate variability (HRV) refers to the physiological variation in the heart rate interval, specifically the R-R interval (RRI), which primarily reflects the functional status of the autonomic nervous system (ANS) and demonstrates the dynamic balance between sympathetic and parasympathetic activities (1, 2). As a crucial biomarker, HRV serves as an essential index for assessing cardiovascular health, stress levels, recovery status, and autonomic regulation (3). Autonomic function is highly susceptible to disruption by various diseases, leading to regulatory imbalances and diverse clinical manifestations. Such autonomic impairment is commonly observed in type 2 diabetes mellitus (T2DM), metabolic syndrome, hypertension, and cardiovascular disorders such as arrhythmias (4). Notably, autonomic dysfunction is particularly prominent in patients with T2DM, where impaired autonomic regulation significantly increases the risk of secondary cardiovascular diseases (5–7). Epidemiological evidence suggests that the prevalence of cardiovascular autonomic neuropathy (CAN) in T2DM patients ranges from 20% to as high as 60%, depending on age, disease duration, and glycemic control, thereby substantially increasing the risk of silent myocardial infarction and mortality (8). Consequently, HRV has become an indispensable parameter for the early detection of autonomic dysfunction and provides critical guidance for designing preventive and therapeutic intervention strategies (9).

Currently, the primary clinical indices of HRV include SDNN, RMSSD, pNN50, SDSD, and LHR (10, 11). Although these traditional indices are widely used, such as in predicting glycemic control (11), they often lack the necessary sensitivity to detect subtle, dynamic changes in complex clinical scenarios. In T2DM and elderly populations, early autonomic neuropathy typically manifests as a progressive, subclinical impairment of regulation that conventional HRV analysis often fails to identify in a timely manner (12). Furthermore, autonomic degeneration in elderly populations is frequently confounded by comorbidities such as hypertension, obesity, and vascular sclerosis (13). The coexistence of these multifaceted factors makes it exceptionally challenging for conventional HRV metrics to accurately reflect the true physiological state when dealing with complex clinical presentations.

A previous study (14) introduced a novel sequence, the TR interval (TRI), which significantly improved the detection sensitivity for populations at high risk of autonomic dysfunction. The traditional TRC sequence incorporates two critical phases of cardiac electrical activity, ventricular depolarization and repolarization (15), thereby offering a more comprehensive assessment of the cardiac cycle and a valuable clinical perspective for identifying early-stage autonomic degradation. However, the traditional TRC framework presents a key technical limitation: its requirement for dual sequential corrections (RRI→TRI→TRC) can lead to error propagation, ultimately compromising computational accuracy and data reliability.

To address this issue, our study proposes an optimized Direct-TRC (DTRC) sequence. This method directly extracts the TR interval (Direct-TRI) from ECG signals and derives the DTRC sequence through a single, streamlined correction step. This approach significantly reduces computational complexity, mitigates mathematical error accumulation, and maintains high precision, allowing the resulting metrics to more closely reflect the true electrophysiological state of the heart. Utilizing this optimized sequence, we calculate key HRV metrics (SDNN, RMSSD, pNN50, SDSD), the low-to-high frequency power ratio (LHR) (16), Poincaré plot indices (short-term scaling ratio SSR) (17), and baroreflex entropy index (BEI) (18). After analysis, these indices can provide more intuitive and accurate assessments of autonomic fluctuations of autonomic nerve function compared with traditional indices. Therefore, the primary objective of this study is to validate the clinical efficacy and sensitivity of the novel single-step DTRC sequence in assessing early autonomic dysfunction and glycemic control-related cardiovascular risks within elderly and T2DM cohorts.

## 2 Parameters and methods

### 2.1 Study design

This study was designed as a retrospective case-control study to evaluate the clinical efficacy of the novel DTRC sequence. The case cohorts comprised patients diagnosed with T2DM (subdivided by glycemic control), while healthy individuals served as the control group. The performance of HRV indices derived from RRI, TRC, and DTRC sequences was systematically compared to assess their sensitivity and accuracy in detecting autonomic nervous function alterations across these distinct cohorts.

### 2.2 Population and settings

#### 2.2.1 Inclusion population

A total of 134 participants, consisting of T2DM patients and healthy individuals, were initially screened. All participants were recruited from the diabetes clinic and the health examination center at Hualien Hospital. Prior to enrollment, written informed consent was obtained from all participants. After screening against the eligibility criteria, 124 subjects were enrolled in the final analysis, comprising 60 T2DM patients and 64 healthy controls.

#### 2.2.2 Inclusion criteria

The clinical diagnosis of T2DM was established based on glycosylated hemoglobin (HbA1c) levels ≥ 6.5%, whereas the control group was confirmed to be free of T2DM with HbA1c levels < 6.5% (19–21). The age of the enrolled participants ranged from 24 to 81 years. Additionally, all participants were required to exhibit normal T-wave morphology on lead II ECG (22, 23).

#### 2.2.3 Exclusion criteria

The study excluded the following populations: professional athletes; individuals with a history of cardiovascular diseases (including coronary artery disease, congestive heart failure, and peripheral arterial disease); subjects with abnormal T-wave morphology in lead II electrocardiograms; individuals taking medications that may interfere with autonomic nervous function (such as beta-blockers, calcium channel blockers, and central antihypertensive agents). Additionally, to minimize confounding influences on HRV, individuals with uncontrolled hypertension and diagnosed thyroid disorders (including hypothyroidism and hyperthyroidism) were strictly excluded.

#### 2.2.4 Group settings

The study divided participants into three groups:

Group 1 comprised 64 healthy individuals (39 males and 25 females) aged 24–79 years, HbA1c < 6.5%.
Group 2 included 35 well blood glucose controlled T2DM patients (25 males and 10 females) aged 35–81 years, 6.5% ≤ HbA1c < 8%.
Group 3 consisted of 25 poor blood glucose controlled T2DM patients (15 males and 10 females) aged 44–73 years, HbA1c ≥ 8%.

### 2.3 Data and parameter calculation

**Figure 1:**
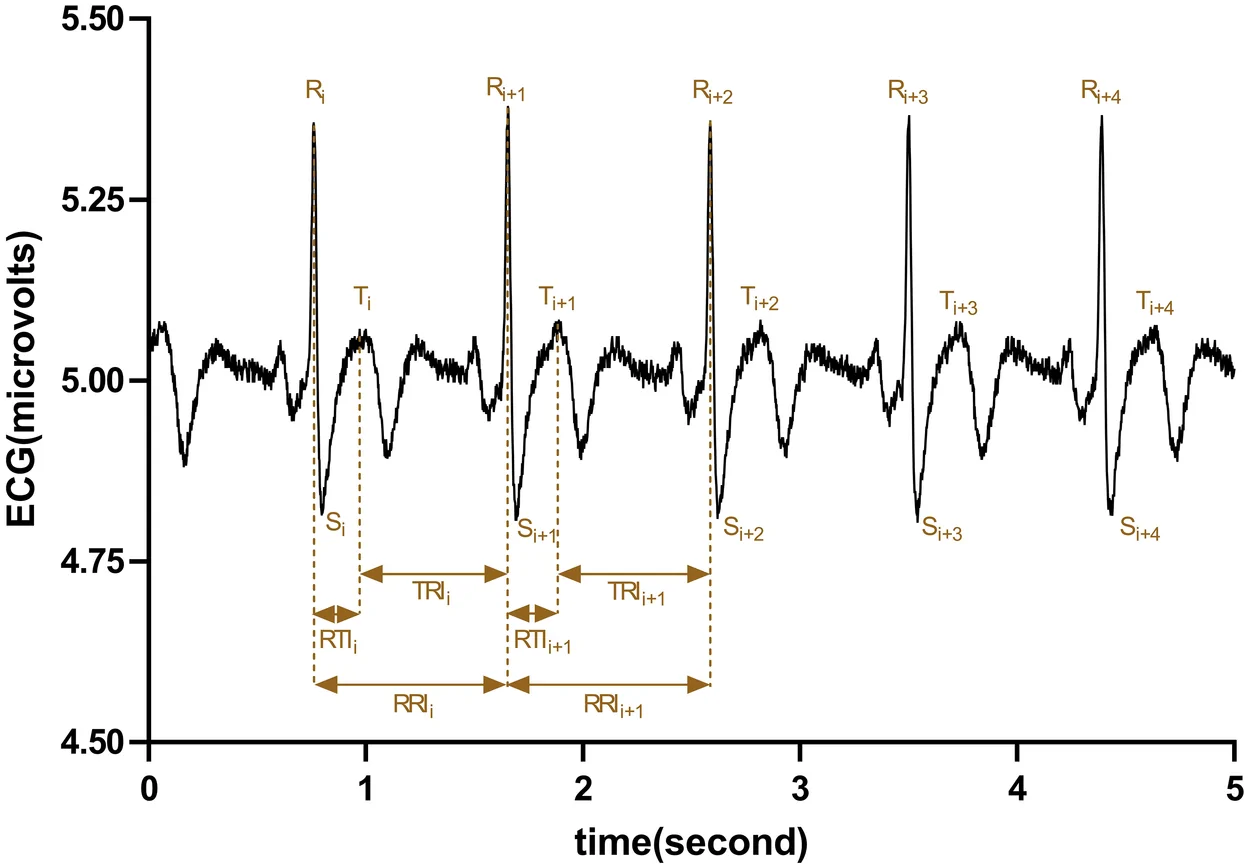
Distinguishing between RTI and TRI in ECG.

The RRI (R-R interval) represents the time interval between two consecutive R-wave peaks in an ECG, representing a complete cardiac cycle. It consists of two components: the RT interval and TR interval (Figure 1). The RT interval is defined as the time interval from the current R-wave peak to the T-wave peak, while the TR interval is defined as the time interval from the current T-wave peak to the next R-wave peak.

To ensure standardized data acquisition, all ECG recordings were performed in accordance with the guidelines for HRV measurement (24). The recordings were consistently scheduled in the morning between 8:00 AM and 11:00 AM in a quiet, temperature-controlled room (22–24 °C) to minimize circadian and environmental variations. Prior to the examination, all participants were instructed to fast for at least 8 h and to avoid alcohol, caffeine, nicotine, and strenuous physical exertion for 24 h.

This study utilized lead II ECG (25) to acquire 30-minute recordings. Because the initial portion of the ECG signals exhibited irregular patterns, the first 99 beats were excluded as a stabilization period to eliminate transient physiological stress responses (e.g., transient anxiety or position-adjustment artifacts) immediately after electrode attachment. Subsequently, the signals from the 100th to the 2000th heartbeat were analyzed, which ensured sufficient data length for nonlinear indicators (like MSE and SSR) while avoiding long-term baseline drift. A custom peak-detection algorithm developed in MATLAB (MathWorks, Natick, MA, USA) was utilized to directly extract the RR interval and TR interval from ECG, yielding the {RRI} and {DTRI} sequences:

$$\left\{RRI\}\;=\;\{RRI_{100},\;RRI_{101},\;\ldots ,\;RRI_{2000}\right\}$$ (1)

$$\left\{DTRI\}\;=\;\{DTRI_{100},\;DTRI_{101},\;\ldots ,\;DTRI_{2000}\right\}$$ (2)

Based on the above two sequences, we corrected the {DTRI} using Bazett’s formula (QTc = QT interval/√RRI, measured in seconds) (26) to obtain the sequence:

$$\{DTRC\}=\frac{\left\{DTRI\right\}}{\sqrt{RRI}}$$ (3)

Prior to parameter calculation, a fourth-order Butterworth zero-phase bandpass filter was applied to denoise the raw ECG signals. This process eliminated low-frequency baseline drift, high-frequency electromyography interference, and powerline noise, while preserving the true phase characteristics of the electrocardiographic waveforms through bidirectional filtering. For massive motion artifacts, abrupt anomalies, and ectopic beats that could not be eliminated by filtering, a mean deviation threshold method combined with manual visual inspection was utilized for precise identification. Subsequently, cubic spline interpolation was performed to correct the local signals, thereby ensuring the continuity and integrity of the ECG time series.

Following artifact rejection, these processed sequences were utilized to calculate HRV indices. These indices were then analyzed to evaluate autonomic nervous function across healthy individuals, middle-aged and elderly adults, and T2DM patients, while systematically examining their corresponding clinical correlations.

### 2.4 HRV indices in Various domains

#### 2.4.1 Time-Domain indices

HRV time domain indices are derived through statistical or geometric methods of calculation and analysis, and are used to assess the overall regulatory capacity of the cardiac autonomic nervous system. The main indices include SDNN, RMSSD, pNN50, and SDSD (27, 28). These parameters reflect overall variability and parasympathetic nervous system modulation, serving as commonly used indices for evaluating autonomic balance and cardiovascular adaptability.

#### 2.4.2 Frequency-Domain Index

LHR (Low to High Frequency Ratio) (29) is the ratio of low-frequency power (LF) to high-frequency power (HF) in HRV frequency-domain analysis, and it is the core frequency-domain index of autonomic nervous system balance. An increase in LHR represents sympathetic dominance (such as during stress and exercise), while a decrease in LHR represents vagal dominance (such as during rest and deep breathing), providing a basis for evaluating autonomic nervous function in clinical and scientific research.

$$LHR=\frac{LF}{HF}$$ (4)

#### 2.4.3 Nonlinear indices

(1) The SSR (Poincaré plot index) (30) is a method that quantifies the geometric characteristics of adjacent RR intervals. It evaluates autonomic nervous function through nonlinear analysis, revealing autonomic regulation details that conventional time-domain or frequency-domain indices cannot provide. The calculation method is as follows:
  $$SD1=\sqrt{var\left(\frac{RR_{n}-RR_{n+i}}{\sqrt{2}}\right)}$$ (5)
  $$SD2=\sqrt{var\left(\frac{RR_{n}+RR_{n+i}}{\sqrt{2}}\right)}$$ (6)
  $$SSR_{i}=\frac{SD1}{SD2}$$ (7)
  $$SSR_{1-10}=\frac{1}{10}\overset{10}{\underset{i=1}{\sum }}\;SSR_{i}$$ (8)

Where *SD1* represents short-term heart rate variability, reflecting instantaneous fluctuations between adjacent RR intervals and serving as a specific index of vagal nerve activity. *SD2* represents long-term heart rate variability, indicating RR interval fluctuations over longer time scales and representing the overall sympathetic-vagal balance (14), while *var* denotes the variance of sample data. *SSR* is the ratio of (5) to (6), quantifying the autonomic balance status and providing critical assessment criteria for cardiovascular diseases, metabolic disorders, and neurological pathologies. Equation (8) calculates the average of SSR_1_ through SSR_10_, preventing any single measurement from becoming excessively large or small due to interference errors, thereby ensuring statistical reliability.

(2) BEI (Baroreflex Entropy Index) is an index measuring baroreflex regulatory function, serving as a novel nonlinear index derived from lead II ECG for autonomic function (AF) assessment in T2DM patients. It reflects the complexity of the human blood pressure control system (18). Higher BEI values indicate superior physiological regulatory capacity, while lower values suggest diminished baroreflex sensitivity and autonomic imbalance. The calculation procedure is as follows:

First, quantize the RRI and Amp sequences to obtain two new sequences:

$$u_{i}=\{\begin{array}{c} 1,Amp(i+1)>Amp(i)\\ 0,Amp(i+1)\leq Amp(i) \end{array},\;v_{i}=\{\begin{array}{c} 1,RRI(i+1)>RRI(i)\\ 0,RRI(i+1)\leq RRI(i) \end{array}$$ (9)

Then, calculate the directional similarity (DS) of the two sequences increasing or decreasing synchronously, as shown in Equation (10):

$$DS_{s}^{m}=\frac{1}{n-m-s+1}\overset{n-m-s+1}{\underset{i=1}{\sum }}\;count(i)$$ (10)

Where *s* denotes the delay number, *m* represents the count of consecutive points in similar intervals between the two sequences, and *count(i)* indicates the number of times both sequences simultaneously increase or decrease for *m* consecutive points within similar intervals.

Finally, calculate the BEI:

$$BEI(m,s)=ln\left[\frac{\sum_{s=1}^{3}\;DS_{s}^{m=2}+\sum_{s=1}^{3}\;DS_{s}^{m=3}+\sum_{s=1}^{3}\;DS_{s}^{m=4}}{\sum_{s=4}^{5}\;DS_{s}^{m=2}+\sum_{s=4}^{5}\;DS_{s}^{m=3}+\sum_{s=4}^{5}\;DS_{s}^{m=4}}\right]$$ (11)

Where ln is the symbol for the natural logarithm operation.

(3) MSE (Multiscale Entropy) is a multidimensional analysis method that quantifies system adaptability by calculating the sample entropy of signals at different time scales, serving as a nonlinear index for assessing physiological signal complexity. Small-scale MSE (scales 1–5) focuses on short-term heart rate fluctuations (reflecting sympathetic nerve function), while large-scale MSE (scales 16–20) emphasizes long-term variability ((reflecting vascular function) (31). MSE can evaluate autonomic nervous function and vascular health status (32), where decreased MSE values typically indicate increased risks of diseases such as diabetes or cardiovascular disorders. Through computation, small-scale entropy (MSE_SS_) and large-scale entropy (MSE_LS_) can be obtained from RRI, TRC, and DTRC sequences.

### 2.5 Statistical analysis

This study employed SPSS (version 26, IBM Corp., Armonk, NY, USA) for statistical analysis. Continuous variables were expressed as mean ± standard deviation (SD), while categorical variables were presented as percentages. One-way ANOVA was used to compare the three different sequences (RRI, TRC and DTRC), with homogeneity of variance tests verifying whether the overall variances were consistent across sequences. Furthermore, LSD *post-hoc* tests were performed to determine pairwise differences among the three groups. To evaluate the diagnostic performance of the indices, receiver operating characteristic (ROC) curve analysis was performed, and the area under the curve (AUC) was calculated to quantify their discriminative ability. Additionally, Pearson correlation analysis was conducted to evaluate the linear relationships and covariation among the RRI, TRC, and DTRC sequences, with the goodness-of-fit assessed using the coefficient of determination (R^2^). Furthermore, to address the significant baseline age discrepancy among the groups and isolate the independent effects of T2DM progression (33), ANCOVA was performed for HRV parameters, utilizing age as a covariate. Subsequently, Bonferroni correction was used to analyze post-hoc comparisons. All statistical tests were two-tailed.

## 3 Results

### 3.1 Basic characteristics and HRV indices of the three groups

Table 1 presents the basic physiological characteristics and HRV parameters of the three participant groups. There were no significant differences among the groups in DBP (*p* = 0.295), HDL (*p* = 0.227), or LDL (*p* = 0.065), whereas other characteristics demonstrated notable group effects. In ANCOVA, the confounding effect of age had an impact on the parameters LHR and SSR (*p* = 0.010, *p* = 0.011), thus, these parameters were statistically adjusted for age. After adjusting for the age factor through ANCOVA, DTRC-based RMSSD (*p* = 0.007), pNN50 (*p* = 0.043), and SDSD (*p* = 0.007) all showed more significant between-group differences compared to the other two sequences, indicating the superiority of DTRC in reflecting vagal nerve activity and autonomic regulation. Furthermore, DTRC also exhibited better statistical performance in LHR, SSR_1–10_, MSE_LS_, and the new index BEI, with *p*-values of 0.016, 0.010, 0.009, and 0.002, respectively.

**Table 1:** Basic physiological characteristics and HRV indices of three groups of personnel.

Parameter	Group 1	Group 2	Group 3	*p* value
total(male/female)	64 (39/25)	35 (25/10)	25 (15/10)	N/A
Age(year)	50.63 ± 12.62	65.17 ± 10.64	59.68 ± 6.96	<0.001
WC(cm)	84.87 ± 10.87	93.85 ± 10.77	95.80 ± 11.03	<0.001
BMI(kg/m2)	24.92 ± 3.46	26.81 ± 5.91	28.51 ± 5.21	0.004
HbA1c(%)	5.88 ± 0.46	6.99 ± 0.59	9.31 ± 1.72	<0.001
FPG(mg/dL)	99.25 ± 12.76	146.3 ± 26.51	200.1 ± 46.68	<0.001
Cholesterol(mg/dL)	170.3 ± 39.67	183.1 ± 38.41	207.0 ± 65.62	0.004
Triglyceride(mg/dL)	102.2 ± 52.40	127.2 ± 53.50	177.5 ± 68.34	<0.001
SBP(mmHg)	118.9 ± 13.36	129.9 ± 20.15	126.3 ± 15.00	0.004
DBP(mmHg)	75.89 ± 9.82	79.17 ± 12.29	78.24 ± 9.46	0.295
HDL(mg/dL)	45.27 ± 14.44	45.34 ± 17.00	39.68 ± 9.97	0.227
LDL(mg/dL)	110.6 ± 35.05	114.7 ± 24.25	131.5 ± 56.00	0.065
SDNN(RRI)	52.2 ± 23.45	61.52 ± 41.16	45.23 ± 33.40	0.131
SDNN(TRC)	56.11 ± 20.95	65.26 ± 35.81	48.31 ± 24.00	0.050
SDNN(DTRC)	95.2 ± 30.94	106.0 ± 37.24	101.3 ± 34.03	0.297
RMSSD(RRI)	42.17 ± 25.22	62.76 ± 57.28	43.83 ± 50.38	0.058
RMSSD(TRC)	48.82 ± 21.66	69.07 ± 49.86	49.93 ± 34.55	0.017
RMSSD(DTRC)	33.31 ± 13.19	45.49 ± 26.69	34.56 ± 15.54	**0**.**007**
pNN50(RRI)	12.43 ± 14.76	16.07 ± 17.7	10.71 ± 19.71	0.424
pNN50(TRC)	20.99 ± 15.19	24.39 ± 19.11	20.15 ± 17.66	0.548
pNN50(DTRC)	9.77 ± 8.01	15.44 ± 13.7	13.11 ± 12.65	**0**.**043**
SDSD(RRI)	42.18 ± 25.22	62.78 ± 57.30	43.84 ± 50.39	0.058
SDSD(TRC)	48.83 ± 21.66	69.09 ± 49.88	49.94 ± 34.56	0.017
SDSD(DTRC)	33.32 ± 13.2	45.50 ± 26.69	34.57 ± 15.55	**0**.**007**
LHR(RRI)	1.39 ± 0.24	1.27 ± 0.28	1.37 ± 0.33	0.095
LHR(TRC)	1.27 ± 0.23	1.15 ± 0.28	1.14 ± 0.30	0.025
LHR(DTRC)	1.19 ± 0.22	1.07 ± 0.27	1.04 ± 0.27	**0**.**016**
SSR_1−10_(RRI)	0.68 ± 0.14	0.73 ± 0.15	0.67 ± 0.20	0.244
SSR_1−10_(TRC)	0.73 ± 0.12	0.8 ± 0.13	0.80 ± 0.14	0.027
SSR_1−10_(DTRC)	0.78 ± 0.12	0.84 ± 0.12	0.85 ± 0.12	**0**.**010**
BEI(RRI)	1.54 ± 0.51	1.30 ± 0.41	1.22 ± 0.51	0.008
BEI(TRC)	1.48 ± 0.36	1.28 ± 0.26	1.25 ± 0.35	0.003
BEI(DTRC)	1.47 ± 0.29	1.30 ± 0.20	1.28 ± 0.29	**0**.**002**
MSE_SS_(RRI)	1.88 ± 0.30	1.72 ± 0.46	1.78 ± 0.37	0.098
MSE_SS_(TRC)	2.09 ± 0.23	2.00 ± 0.40	2.12 ± 0.31	0.243
MSE_SS_(DTRC)	2.18 ± 0.23	2.11 ± 0.38	2.19 ± 0.33	0.442
MSE_LS_(RRI)	2.13 ± 0.37	1.94 ± 0.44	1.95 ± 0.43	0.031
MSE_LS_(TRC)	2.14 ± 0.38	1.91 ± 0.38	2.01 ± 0.39	0.017
MSE_LS_(DTRC)	2.09 ± 0.35	1.86 ± 0.38	1.91 ± 0.39	**0**.**009**

Group 1: Healthy individuals; Group 2: T2DM patients with good glycemic control (6.5%≤HbA1c < 8%); Group 3: T2DM patients with poor glycemic control (HbA1c ≥ 8%). WC, waist circumference; BMI, body mass index; HbA1c, glycated hemoglobin; FPG, fasting plasma glucose; SBP, systolic blood pressure; DBP, diastolic blood pressure; HDL, high-density lipoprotein; LDL, low-density lipoprotein; SDNN, standard deviation of normal RR intervals; RMSSD, root mean square of successive RR interval differences; pNN50, percentage of adjacent normal RR intervals differing by >50 ms; SDSD, standard deviation of differences between adjacent normal RR intervals; LHR, low-to-high frequency power ratio; SSR_1−10_, poincaré plot indices; BEI, baroreflex entropy index; MSE_SS_ and MSE_LS_, multiscale entropy indices for small and large scales. *p*-value <0.05 was considered statistically significant. Bold values indicate that the p-value of this DTRC-Based index are superior to those of the RRI-Based and TRC-Based indices.

In the LSD *post-hoc* tests, pNN50 differed significantly only between Group 1 and Group 2 (*p* = 0.014). In contrast, RMSSD and SDSD showed significant differences both between Group 1 and Group 2 (*p* = 0.002) and between Group 2 and Group 3 (*p* = 0.025), suggesting a progressive decline in parasympathetic activity in patients with T2DM. For LHR, SSR_1–10_, MSE_LS_, and BEI, significant differences were consistently observed between Group 1 and the other two groups, indicating impairments in autonomic balance, as well as vascular and blood pressure regulation, in T2DM patients.

These findings demonstrate that DTRC-based HRV indices not only exhibit improved sensitivity but also enhance the discriminative capacity of HRV indices, thereby better reflecting autonomic function and vascular health status in diabetic and elderly populations.

### 3.2 Correlation analysis

**Figure 2:**
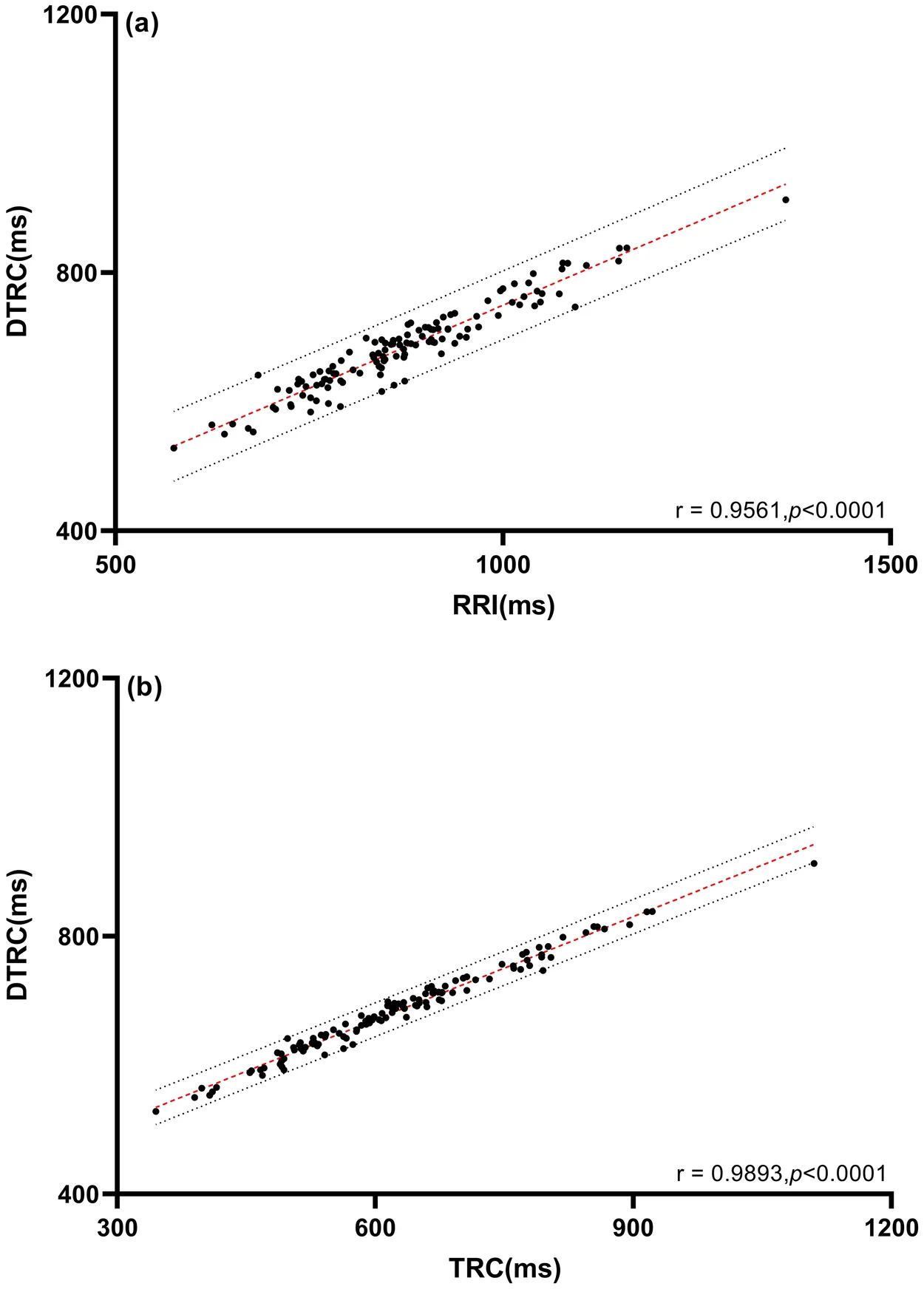
**(a)** is the correlation analysis between RRI and DTRC. **(b)** is the correlation analysis between TRC and DTRC, where r represents Pearson’s correlation coefficient and *p*-values <0.05 indicate statistical significance.

To evaluate the consistency of DTRC-based HRV indices, this study conducted correlation analyses between RRI and DTRC, as well as between TRC and DTRC, to measure their degree of association. The correlation analysis yielded coefficient of determination (R^2^) values (representing the goodness-of-fit of linear regression model) of 0.9141 for RRI-DTRC and 0.9787 for TRC-DTRC. Figure 2 demonstrates significant positive correlations for both RRI-DTRC (r = 0.9561, *p* < 0.001) and TRC-DTRC (r = 0.9893, *p* < 0.001). These findings confirm that the strong associations among the three sequences are highly unlikely to be caused by random errors, demonstrating a robust covariant relationship.

### 3.3 Ternary plot of FPG, HbA1c and DTRC-based HRV indices

**Figure 3:**
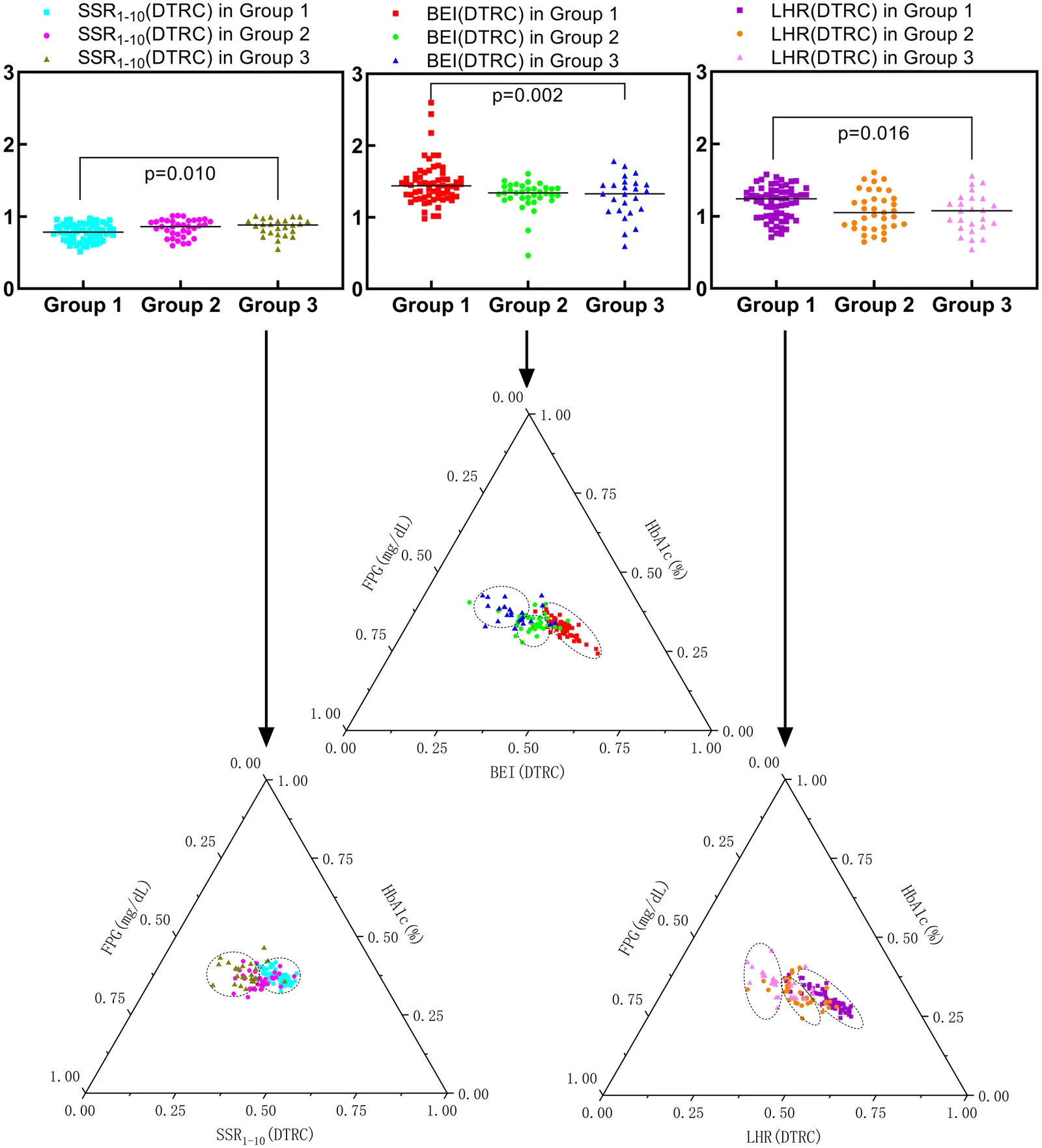
The ternary plot evaluates the separation capability of DTRC-based HRV indices across groups and reveals the influence of FPG and HbA1c on HRV indices.

This study employed ternary plot visualization to directly compare the discriminative capacity of RRI-based and DTRC-based HRV indices across the three groups. Fasting plasma glucose (FPG), glycated hemoglobin (HbA1c), and HRV indices (SSR_1–10_, LHR, BEI) were selected as core variables not only due to their significant correlations with diabetes (all showing statistical differences in Table 1), but also because they collectively reflect the interaction between glucose metabolism and autonomic function. In Figure 3, the three axes of the ternary plot respectively represent standardized proportions (0%–100%) of FPG, HbA1c, and composite HRV indices. This visualization method simultaneously displays the relative contributions of three variables through spatial distribution of points in a two-dimensional plane, revealing characteristic HRV deviations when FPG or HbA1c dominates, while intuitively presenting distribution differences among the three groups through data point clustering.

The results indicate that the three HRV indices based on the DTRC sequence show significant statistical differences (The *p*-values of the three indices from left to right are 0.010, 0.002, and 0.016, respectively), which can clearly distinguish BEI(DTRC) and LHR(DTRC) among the three groups. Regarding SSR_1–10_(DTRC), a clear distinction is primarily observed between Group 1 and Group 3, whereas the distribution of Group 2 is less concentrated. Overall, the HRV indices based on the DTRC sequence provide good visual discrimination for inter-group differences.

### 3.4 Comparative analysis of ROC curves

**Figure 4:**
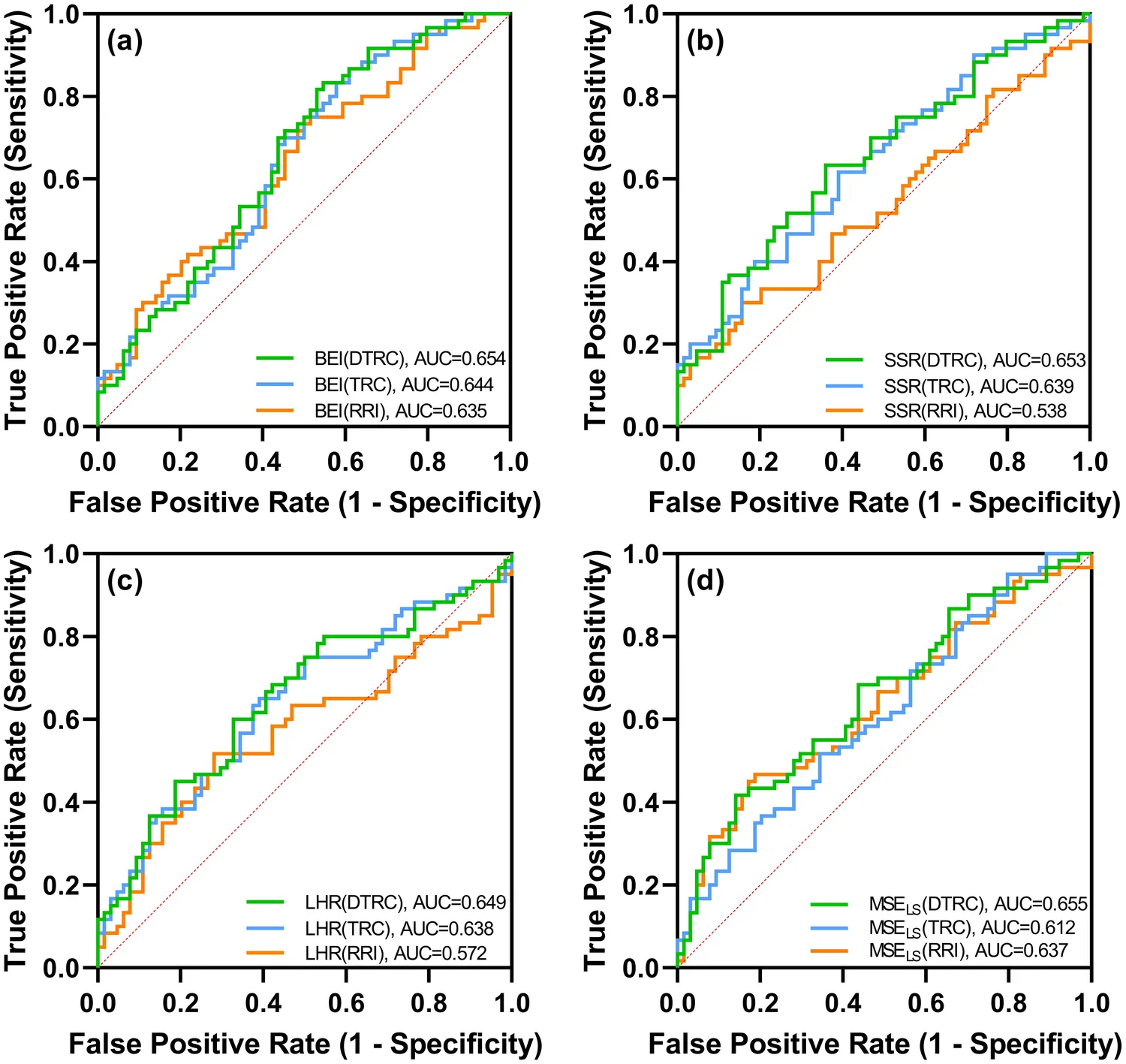
ROC curves of HRV indices based on RRI, TRC, and DTRC sequences. The indices corresponding to **(a)**, **(b)**, **(c)**, and **(d)** are BEI, SSR, LHR, and MSE_LS_, respectively. Due to the lack of significant differences in MSE_SS_, ROC curve analysis will not be performed on it.

 Figure 4 illustrates the comparison of ROC curves for each index when using RRI, TRC, and DTRC sequences as input parameters. The results demonstrate that the AUC value of each DTRC-based index is the highest among the three configurations, indicating that the HRV indices is more sensitive to DTRC sequences and has higher discrimination. The highest increase was observed in the AUC value of MSE_LS_(DTRC), which was 0.043 higher than that of MSE_LS_(TRC), indicating that the DTRC sequence has superior discriminative ability in vascular function.

## 4 Discussion

This study confirms the clinical value of the DTRC sequence in HRV assessment, demonstrating particular advantages for evaluating autonomic function in diabetic and elderly populations. While the conventional RRI sequence is limited to reflecting heartbeat interval variations, the TRC sequence overcomes this constraint, and the DTRC further significantly enhances detection sensitivity in complex pathological conditions (for example, T2DM and age-related autonomic decline). Results show that DTRC-based HRV indices (including LHR, SSR_1–10_, and BEI) exhibit markedly superior discriminative performance among the three groups compared to traditional RRI and TRC indices (Table 1). Specifically, DTRC significantly enhanced detection sensitivity for multiple key parameters (RMSSD, pNN50, SDSD, LHR, SSR_1–10_, BEI and MSE_LS_; *p* = 0.007, 0.043, 0.007, 0.016, 0.010, 0.002 and 0.009, respectively), although some indices like SDNN showed no statistical significance. This discrepancy can be explained by their distinct physiological and mathematical properties (34). SDNN represents the overall standard deviation of the entire recording, which is primarily influenced by low-frequency, long-term fluctuations governed by both the sympathetic and parasympathetic nervous systems. Conversely, RMSSD measures the root mean square of successive differences, reflecting short-term, high-frequency heart rate variations mediated exclusively by parasympathetic activity. The single-step DTRC algorithm is optimized to neutralize slow-moving baseline wander, respiratory modulations, and steady-state repolarization drifts. By filtering out these low-frequency noise overlays, the high-frequency micro-fluctuations captured by RMSSD are isolated and amplified, revealing the subtle vagal withdrawal characteristic of early diabetic neuropathy. On the other hand, the removal of these slow baseline trends naturally compresses the macro-variability measured by SDNN, explaining why this parameter did not benefit from the DTRC correction.

Several limitations of this study should be explicitly acknowledged. First, the cross-sectional design of our study inherently prevents the establishment of a causal relationship between autonomic dysfunction and the progression stages of T2DM. Longitudinal cohort studies are highly warranted in the future to track the prognostic value of DTRC indices over time. Second, a structural limitation of this study lies in the exclusive reliance on Lead II ECG recordings. Although Lead II is widely considered the gold standard for R-wave detection and basic interval segmentations, it provides a unidirectional projection of cardiac electrical vectors. Consequently, it may fail to fully capture three-dimensional, complex spatial vectorial changes in cardiac repolarization, specifically during the T-wave interval, which could be more comprehensively characterized by multi-lead configurations (e.g., 12-lead ECG) (35). Future studies should incorporate multi-lead data to evaluate whether spatial vectorial indices further enhance the sensitivity of DTRC indices. Lastly, while we strictly excluded medications with direct autonomic-influencing properties, the potential confounding influence of long-term background diabetic therapies cannot be entirely dismissed. For example, Metformin may exhibit neuroprotective properties and modulate vagal activity, while SGLT2 inhibitors can reduce sympathetic overactivation. Therefore, future studies with drug-naive cohorts are essential to isolate the pure, independent interaction between glycemic control, diabetic neuropathy, and DTRC indices.

Additionally, it is worth noting that although the HRV index based on DTRC showed statistical differences in inter-group comparisons (Table 1), the discriminative power of their ROC curve analysis (Figure 4) still needs to be improved and may require more samples for validation. To bridge this gap and transition DTRC from a screening tool to a robust clinical diagnostic system, several refinements are necessary. First, rather than relying on isolated univariate metrics, future clinical models should integrate multi-parametric fusion frameworks that combine DTRC temporal features with frequency-domain and non-linear complexity indices. Second, incorporating these indices into machine learning classifiers (such as support vector machines or deep neural networks) trained on larger, multi-center cohorts could drastically enhance classification boundaries. Lastly, pairing DTRC parameters with established clinical biomarkers, such as HbA1c trajectories and the duration of diabetes, will likely yield the high-precision diagnostic accuracy required for routine clinical implementation.

To the best of our knowledge, this is the first study to introduce and systematically evaluate the single-step DTRC sequence in HRV analysis. While previous studies have utilized Bazett's formula to correct the TRI sequence (generating the TRC sequence) to evaluate diabetic and age-related autonomic decline (14), or applied lead II ECG-derived entropy for autonomic screening (18), those approaches involved a multi-step baseline correction process. The improved performance of the newly proposed DTRC-based indices likely stems from algorithmic optimization compared to TRC. By reducing one correction step and its associated errors, DTRC more accurately reflects original ECG signal characteristics, thereby minimizing systematic errors and better representing patients’ physiological states. Furthermore, this technology enhanced DTRC with greater sensitivity to baroreflex function, as validated by BEI measurements. As a specialized index for evaluating baroreflex function, the DTRC-based BEI calculation demonstrates significantly enhanced detection capability under pathological conditions. This advantage originates from DTRC's superior precision in capturing cardiovascular regulatory abnormalities during baroreflex arc impairment, these abnormalities often underestimated in conventional HRV analysis (36).

The results of this study not only verify the previous research findings on the characteristics of HRV in the elderly and diabetic populations, but also achieve an important breakthrough in methodology. Traditional RRI-based HRV indices demonstrate notable limitations in discriminating these populations, particularly in detecting diabetic autonomic neuropathy: they cannot comprehensively capture the multidimensional abnormalities in autonomic regulation among diabetics, and show no statistically significant differences in intergroup comparisons. These limitations may stem from the RRI sequence's insufficient sensitivity to key physiological signals like baroreflex activity, making them inadequate for detecting early-stage autonomic dysfunction in diabetes.

From a clinical perspective, the DTRC sequence possesses high practical utility due to its reliance on standard lead II ECG data without requiring invasive monitoring or complex multi-channel systems. This algorithmic framework can be seamlessly integrated into existing clinical Holter monitors, digital health platforms, and wearable smart devices. In clinical practice, it can serve as an automated, cost-effective screening tool during routine health check-ups to identify T2DM patients or elderly individuals presenting with subclinical cardiac autonomic neuropathy (CAN). This facilitates timely lifestyle interventions or pharmaceutical management before irreversible cardiovascular damage occurs. To successfully translate these findings into clinical screening protocols and smart wearable health monitoring devices, several concrete steps must be pursued: (1) compiling the DTRC single-step algorithm into lightweight, embedded libraries compatible with ultra-low-power microcontrollers used in smart bands, (2) developing robust, real-time noise-canceling filters to handle motion artifacts in dynamic wearable environments, (3) establishing a cloud-based automated screening diagnostic pipeline that seamlessly transmits wearable-derived DTRC metrics to electronic health records systems, and (4) validating these metrics in prospective, large-scale clinical trials to establish age- and sex-stratified healthy reference ranges.

## 5 Conclusion

This study introduces an innovative cardiovascular health assessment method, with its core technology being the application of DTRC sequences for HRV indices analysis. This approach overcomes the limitations of traditional RRI-based HRV indices and demonstrates superior detection performance compared to TRC-based HRV indices. Our data confirm that DTRC not only performs well with conventional HRV indices (RMSSD, pNN50 and SDSD) but also shows significant statistical differences in advanced HRV indices (LHR, SSR_1–10_, BEI and MSE_LS_). Moreover, DTRC-based HRV indices exhibit several outstanding characteristics: significantly improved detection sensitivity, remarkable clinical utility, and the ability to identify autonomic nervous system features in elderly and diabetic populations that conventional methods cannot detect. This innovation not only enhances the existing HRV analysis system but also provides a more precise alternative for T2DM research, demonstrating substantial clinical translational significance.

## Data Availability

The raw data supporting the conclusions of this article will be made available by the authors, without undue reservation.
